# Knowledge of end-of-life wishes by physicians and family caregivers in cancer patients

**DOI:** 10.1186/s12904-021-00823-1

**Published:** 2021-09-10

**Authors:** Jose A Calvache, Socorro Moreno, Gillian Prue, Joanne Reid, Sam H Ahmedzai, Angelica Arango-Gutierrez, Liliana Ardila, Lucia I Arroyo, Esther de Vries

**Affiliations:** 1 Department of Anesthesiology, Universidad del Cauca, Popayán, Colombia; 2 Department of Anesthesiology, Erasmus MC University Medical Center Rotterdam, Rotterdam, The Netherlands; 3 Department of Clinical Epidemiology and Biostatistics, Pontificia Universidad Javeriana, Bogotá, Colombia; 4 Reader in Chronic Illness, School of Nursing and Midwifery, Queen’s University Belfast, Belfast, UK; 5 Professor of Cancer and Palliative Care, School of Nursing and Midwifery, Queen’s University Belfast, Belfast, UK; 6 Academic Unit of Supportive Care, Section of Oncology, School of Medicine and Biomedical Sciences, University of Sheffield, Royal Hallamshire Hospital, Sheffield, UK; 7 Clinical Epidemiology, Pontificia Universidad Javeriana, Bogotá, Colombia; 8 National Cancer Institute, Bogotá, Colombia; 9 Departamento de Fonoaudiología, Universidad del Cauca, Popayan, Colombia; 10 MPH programme Public Health, Pontificia Universidad Javeriana Cali, Cali, Colombia

**Keywords:** End of life, Cancer, Communication, Advance Directives, Decision Making

## Abstract

**Objectives:**

To describe communication regarding cancer patient’s end-of-life (EoL) wishes by physicians and family caregivers.

**Methods:**

An online questionnaire and telephone-based surveys were performed with physicians and family caregivers respectively in three teaching hospitals in Colombia which had been involved in the EoL care of cancer patients.

**Results:**

For 138 deceased patients we obtained responses from physicians and family caregivers. In 32 % physicians reported they spoke to the caregiver and in 17 % with the patient regarding EoL decisions. In most cases lacking a conversation, physicians indicated the treatment option was “clearly the best for the patient” or that it was “not necessary to discuss treatment with the patient”.

Twenty-six percent of the caregivers indicated that someone from the medical team spoke with the patient about treatment, and in 67% who had a conversation, caregivers felt that the provided information was unclear or incomplete. Physicians and family caregivers were aware if the patient had any advance care directive in 6% and 26% of cases, respectively, with low absolute agreement (34%).

**Conclusions:**

There is a lack of open conversation regarding EoL in patients with advanced cancer with their physicians and family caregivers in Colombia. Communication strategies are urgently needed.

**Supplementary Information:**

The online version contains supplementary material available at 10.1186/s12904-021-00823-1.

## Background

As Colombia´s population is rapidly ageing, mortality patterns shift from being dominated by unnatural causes and communicable diseases towards dominance by prevalent chronic diseases [1]. The growing number of patients with chronic diseases has led to increasing consciousness in society and the medical community that end-of-life (EoL) care for patients with chronic conditions may involve complex decision-making processes [2].

One of the main goals of palliative care and EoL care is to reduce suffering [3]. Suffering depends on very individual factors and therefore it is impossible to provide high-quality palliative care without effective communication with patients, families, and caregivers regarding their needs. In decision-making in this stage of life, communication may act as a facilitator or as an obstacle [4, 5]. Formal conversations about advanced care directives (ACD) are increasingly used in high-income settings and seem to improve the quality of life and quality of dying by prioritizing goals and reducing unnecessary treatments [6–8]. In addition, they assist in the decision-making process and reduce difficulties in making such decisions for the healthcare professionals [8].

Growing literature supports that extensive EoL discussions are associated with lower rates of aggressive interventions, lower health care costs, and better quality of EoL for patients [9, 10]. In contrast, poor quality communication can result in futile continuation of life support, leading to futile, non-beneficial treatment measures that can exacerbate feelings of distress and frustration in patients, family members, and caregivers [11, 12].

Patients report that several aspects of communication could be improved during EoL care, such as the provision of information, emotional support, and being treated with respect [13]. Patients have reported that their wishes related to health care at the EoL are often not met [14–16], frequently resulting in discordant care [17]. A lack of appropriate information provision from physicians and/or poor patient-physician communication may explain these outcomes. In addition, in the main cultural groups in Colombia, there is a strong taboo on talking about death and dying. This can lead to sub-optimal decision-making processes, poor communication, potentially inappropriate care and symptom management [18] and suboptimal quality of life at the EoL and quality of dying [19]. In Colombia, religious dilemmas, ethical or legal concerns may also play an important role [20].

In Colombia, palliative care has been regulated since 2014 and discussions regarding actively ending life have resulted in euthanasia regulation [21, 22]. It is unknown whether and how actively Colombian patients communicate with their physicians and caregivers regarding their wishes and needs. Previous studies indicate substantial levels of intensive cancer treatments very close to the EoL [12], whilst physicians report little communication regarding EoL decisions with patients and caregivers [23], low rates of formally formulated ACD (personal communication), and a low level of integration between oncology and palliative care [24]. In this study, we aimed to describe knowledge regarding cancer patient’s wishes at the EoL by their treating physicians and family caregivers.

## Methods

 We designed an exploratory and descriptive cross-sectional survey including physicians and family caregivers of cancer patients at the EoL. The survey was delivered online for the physicians and by telephone for family caregivers of patients who attended one of three participating teaching hospitals between May 2019 and May 2020: Instituto Nacional de Cancerología Bogotá (INC), Hospital Universitario San Ignacio Bogotá (HUSI), and Hospital Universitario San José Popayán (HUSJ). All three have specialized oncology services and palliative care teams. The first two are in Bogota, the INC being a specialized and public cancer referral hospital, attending over 7000 new patients per year, and HUSI being a non-profit, tertiary hospital. HUSJ is a public hospital in a Colombian province in the city of Popayán, attending the urban population (> 300,000 inhabitants) and a largely rural area, including several indigenous populations.

Colombia has a mandatory “universal” national social insurance system, including two main insurance structures. The first is contributory, which is financed by payroll contributions and secondly, a subsidized system for the most impoverished population by general taxation. Also, unique, and exceptional groups consist of specific government workers (public teachers, military, police, and state oil company) who have their schemes [25]. The three participating hospitals attend patients affiliated with the different systems: HUSI mainly treats patients covered under the contributory scheme. INC and HUSJ treat patients under both systems and patients from unique and exceptional schemes.

Nurses and physicians identified oncological patients, notified the research team when a cancer patient with an estimated life expectancy of three months or less was seen at the outpatient clinic, emergency department or inpatient wards of the participating hospitals. This prognostic assessment was based on functional scales (ECOG Scale of Performance Status or Karnofsky index) and progressive deterioration of the patients. When these patients deceased, a researcher obtained basic information and invited the attending physicians who had been closely involved in the patient’s EoL care during their last hospitalization stay, to participate within the study. The physician was asked to forward the survey to a colleague if they felt the colleague had a better understanding of the decisions surrounding the patient. As a result of this process, it is possible that some physicians answered the survey for more than one patient.

Family caregivers of the deceased patients were identified based on the medical records (Colombian medical records specify a caregiver’s data). At least two months after the date of death of the patient, research assistants from each hospital contacted the family members by telephone, explained the objectives of the study, and asked them if they would be willing to answer some questions regarding the care provided to the patient during the last phase of life. If they consented, an appointment for a telephone survey was made, and family caregivers provided verbal informed consent, which was audio recorded. A substantial proportion of the Colombian population is functionally illiterate, particularly among the elderly, where cancer and providing care for relatives with cancer are more common [26], therefore we considered this method of data collection appropriate and ethical for this sample.

The physicians received a link to the online survey, which focused on the characteristics of the EoL decision-making that preceded the death of the patient involved, details are provided elsewhere (see Additional file 1), [27–29]. When a decision had a potential life-shortening effect, physicians were asked if they had spoken to the patient and/or family caregivers regarding this potential effect of the treatment decision. Physicians were also asked if they knew the patient had an ACD.

Among the family caregivers, the telephone survey measured their level of involvement in the care of the patient, demographic information, and structured questions regarding the type of care received, information received by the healthcare providers, conversations with the healthcare providers, and if the patient had an ACD or had expressed wishes or preferences regarding treatment and other issues regarding the EoL (see Additional file 2). The survey contained a series of questions considered in the original CEQUEL instrument (Caregiver Evaluation of the Quality of End-Of-Life Care (CEQUEL) Instrument) [30]. Face validity was evaluated by discussion both by the research team and some healthcare providers; the research team provided three rounds of written, individual feedback on the survey. The instrument was pilot tested, initially on a few anonymous volunteers, all bereaved family members of cancer patients, and in a later version on the first two participants, who did not show any difficulty in understanding hence no changes were needed. Three members of our research team (AA, LA, LIA) were trained by a psychologist in the procedure of the telephone survey to use a systematic structured procedure, e.g., how to give further explanation if a respondent does not know how to interpret the question.

 The study protocol was approved by the research ethics committees at Pontificia Universidad Javeriana (number FM-CIE-0086-17) and NCI (Instituto Nacional de Cancerología, number INT-OFI-03581-2019). The physicians answered the survey questions anonymously – to further guarantee anonymity, no information on specialization, age, sex, or years or experience of the participating physician was collected. The physicians were informed that completing the survey implied consent to participate in the study. The survey included very sensitive questions regarding complex decisions, and it was essential to ensure the anonymity of participating physicians. Each participating institution had a list of the coding and identifying information of the patients, kept by the research assistants, who had no access to the databases. This linking information was destroyed after the data had been collected to ensure anonymity; the researchers never had access to the patients’ or physicians’ identifying information.

 Family members were contacted by telephone to ask for their consent to participate in the study and provided verbal consent. We offered confidentiality of the data and guaranteed an anonymized analysis of their responses. The identification data were used to link the physician and family member information to guarantee that the agreement analysis occurred at the individual level. All methods were performed in accordance with the relevant local and international guidelines and regulations.

Analyses were conducted using SPSS Version 25 (IBM). General characteristics of the surveys were summarized using absolute frequencies, proportions, means, medians, and interquartile ranges (IQR). Absolute agreement (or proportion of overall agreement) between physicians and caregivers’ responses was calculated for the following three questions (1) Did the patient receive palliative care?; (2) Did the patient receive treatment for pain or other symptoms?; and (3) Did the patient have any explicit advance care directive? This was calculated by adding the number of affirmative and negative responses in which physicians and caregivers agreed, divided by the total number of ratings [31]. In addition, Cohen’s kappa coefficient was calculated to the last question.

## Results

 We obtained responses from 261 physicians and 176 caregivers of 341 identified patients (response rate 76.5 and 51.6 % respectively); this led to 138 cases were both physicians and caregivers of the same patient participated. Most physicians (95 %) confirmed they were the treating physicians and most of them described the death of the patient as expected (*n* = 124, 90 %). All caregivers were family members: partner of the deceased (*n *= 26, 19 %), parents (*n* = 6, 4 %), siblings (*n* = 23, 17 %), adult children (*n* = 72, 52 %), and other family members (*n* = 14, 10 %). Half of the caregivers had lived with the patient; most had an educational level of high school or lower (66 %); 91 % described themselves as “very involved in the patient care”.

The median time between death and the physicians completing the survey was nine days (IQR 6–20 days). The median time between death and the telephone survey with caregivers was 23.8 weeks (IQR 22.1–27 weeks).

Table 1 presents the distribution of patients’ general characteristics – mean age was 61.5 years (SD 15.3), half were female, and most had died in hospital (86 %).

**Table 1:** General characteristics of patients

Variable	Total ***n***=138 n (%)	HUSI^a^ ***n***=43 n (%)	INC^a^ ***n***=83 n (%)	HUSJ^a^ ***n***=12 n (%)
Age (mean ± SD, median [IQR])	61.5 ± 15.3	64.7 ± 15.3	58.9 ± 14.5	67.9 ± 18
	64 [51-73]	67.5 [60.5-75.5]	59 [50-69]	70 [50.7-85.7]
Female gender	72 (48)	15 (35)	49 (59)	8 (67)
Top three cancer diagnosis	Gastric 26 (19)	Gastric 8 (19)	Gastric 15 (18)	Gastric 3 (25)
	Colorectal 14 (10)	Colorectal 5 (12)	Colorectal 9 (11)	Breast 2 (17)
	Breast 13 (9)	Breast 3 (7)	Breast 8 (10)	Cervical 2 (17)
Health care insurance	Contributive 70 (51)	Contributive 42 (98)	Contributive 24 (29)	Contributive 4 (33)
	Subsidized 56 (41)	Subsidized 0 (0)	Subsidized 49 (59)	Subsidized 7 (58)
	Other 12 (8)	Other 1 (2)	Other 10 (12)	Other 1 (8)

^a^*HUSI* Hospital Universitario San Ignacio, *INC* Instituto Nacional de Cancerología, *HUSJ* Hospital Universitario San José

Physicians and caregivers agreed in most cases that the patient had received drugs to control pain and other severe symptoms (physicians: *n* = 123, 89 %; caregivers *n* = 125, 91 % - absolute agreement physicians – caregivers 83 % (Table 2).

**Table 2:** Affirmative responses by physicians and caregivers and agreement between them regarding end-of-life questions

Question	Affirmative response by physicians n (%)	Affirmative answer by caregivers n (%)	Proportion of absolute agreement (%)^a^
Did the patient receive palliative care?	120 (87 %)	122 (88 %)	78 %
Did the patient receive treatment for pain or other symptoms?	123 (89 %)	125 (91 %)	83 %
Did the patient have any explicit advance care directive?	8 (6 %)	36 (26 %)	34 %

^a^Absolute agreement was calculated by adding the number of affirmative and negative responses in which physicians and caregivers agree, divided by the total number of ratings [31]

In 44 cases (32 %), physicians reported that they spoke to the caregiver regarding the potential effect of hastening the patient’s death because of the decisions made in the last phase of life. In 11 of those cases (25 %), not postponing the patient’s death was requested by caregiver. In 24 cases (17 %), physicians responded that they spoke directly with the patient about the potential hastening of their death because of the intervention. Three patients requested to hasten their EoL, but their requests were not fulfilled. Physicians indicated they agreed regarding the non-use of resuscitation manoeuvres at the EoL with the patient in 26 cases, with patients’ family members in 80, and other caregivers in 14 cases. There was disagreement on this matter in 9 cases (20 %).

In the absence of a conversation about interventions at the EoL, physicians indicated mostly they had not discussed this because the treatment option was “clearly the best for the patient” (*n* = 30, 22 %) or that it was “not necessary to discuss treatment with the patient” (*n* = 18, 13 %).

Few caregivers (*n* = 36, 26 %) indicated that someone from the medical team spoke with the patient about medical treatment preferences during the last week of life; caregivers reported no conversation at all in 71 patients (51 %) and, in 12 patients (9 %) they did not know. In 23 of the 36 patients who had a conversation (67 %), caregivers felt that the information provided by the medical team was unclear or incomplete. Caregivers perceived those medical interventions had prolonged patients´ life in 46 cases, and in 32 of these, the caregiver felt that this prolongation had increased the patient’s suffering.

Physicians reported eight patients had ACD (6 %), of which three were formally formulated. For most cases (*n* = 90, 65 %), physicians reported patients did not have any ACD and in 40 cases (29 %) physicians did not know if the patient had any ACD. Caregivers of 36 patients indicated they knew the patient had their wishes described in an ACD (26 %) (10 formally, 26 informally formulated). Most caregivers (*n* = 74, 54 %) indicated that their relative had no ACD or formally formulated requests, and 28 reported they did not know if the patient had formulated their wishes (20 %). The proportion of absolute agreement between physicians and caregivers, on whether the patient did have an ACD or not was 34 % (Table 2) and Cohen’s kappa coefficient was 0.18. Most of the patients of whom physicians knew they had an ACD were affiliated to the contributive insurance regime (7 out of 8 patients, 88 %). Also, caregivers of patients of the contributive system were more aware about the existence of the ACD of their relative (21 out of 36, 59 %).

## Discussion

Our results show a lack of communication and awareness among physicians and even family caregivers regarding the patient’s EoL preferences. Our study design cannot elucidate the reasons behind this phenomenon, but it is likely that neither physicians nor family caregivers had explicit discussion with the patient regarding preferences, wishes and fears regarding the EoL [2].

The low agreement (34 %) between physicians and caregivers regarding the existence of ACD indicate that, even though only a minority of terminally ill cancer patients had expressed such directives, the communication of those directives was limited – often either a physician or a caregiver (29 and 20 % respectively) (or perhaps both) were not aware if any directive or request existed. Most patients in our study died in hospital, and therefore it would be expected they were in close contact with their physicians, yet the knowledge regarding ACD was still low; probably because physicians and family caregivers are often more focused on physical symptom management, leaving attention to feelings and desires as lower priority.

The increase in technological possibilities of treatment and care has led, in many countries, to regulation regarding patients’ rights to refuse treatment or have shared decision-making on whether life-sustaining therapies will be used in their care [32]. As palliative and EoL care aim to relieve suffering and optimize quality of life, it is important to know if and how patients suffer and what matters to them. Shared decision making requires effective and empathic communication between formal and informal caregivers and the patients [2, 33]. It has been shown that patients with advanced cancer prefer early and open communication about EoL topics [34]. Among other outcomes, poor communication leads to significant misunderstandings by patients and caregivers regarding the nature and seriousness of the disease, treatment, and prognosis.

Our results show a general lack of such conversations: physicians demonstrate a generally paternalistic attitude, where they could decide on what would be best for the patients, which has been commonly noted in similar scenarios [35]. Caregivers were on occasion unaware if patients had an ACD. Additional information from a qualitative study -executed in parallel with this study- suggests that family caregivers often felt that either they or the patients were not optimally heard, or that their preferences were not taken completely into account [36]. Studies have shown a wide variability of knowledge about ACD formulated by patients, and it is clear that there are large disparities in ACD completion, highlighting the need for education about their role in facilitating EoL decisions [37]. One of the frequently reported reasons for not having an ACD is lack of awareness of this option [37–40]. It is possible that this played a significant role in our population and thus highlights the need for education about their role in facilitating EoL care. Finally, other studies have elucidated some potential determinants of ACD knowledge including older age, educational level, and higher income [37].

Study limitations include the potential of selection bias: physicians may have been more prone to decline participation for patients who died outside of hospital, as they would be less informed about those patients’ EoL issues. Similarly, family caregivers´ own experiences may have influenced their decision to participate. Time between death of the patient and survey of the caregiver (approximately 24 weeks) may have introduced recall bias. However, the time frame of 2–12 months has been shown by previous research to allow bereaved caregivers to remember their experience yet giving sufficient time to the participant for grieving [41] and considers the ethical concerns when approaching bereaved caregivers [42]. Telephone surveys were considered necessary because a substantial proportion of the Colombian population is functionally illiterate. It was impossible to reach some caregivers, either because of erroneous telephone numbers (7 %) or because they never answered the telephone (22 %); 91 % of caregivers who were reached decided to participate. Finally, absolute agreement of responses between physicians and caregivers is informative and useful, but it does not distinguish between agreement on positive ratings and agreement on negative ratings. However, for this question, we also calculated Cohen’s kappa coefficient resulting in a very low agreement value.

Advance care planning conversations help individuals to exercise autonomy and make informed decisions about their care. People who lack the knowledge to have EoL concerns or discussions or about the role of ACDs in facilitating EOL decisions, may represent potential targets for intervention. Patient’s psychosocial experience, symptom management, treatment decisions, and quality of life are associated with communication in cancer care that should be considered and prioritized in any agenda [43] including EoL care in low- and middle-income countries.

There are several educational resources than can be adapted, adjusted, and culturally contextualized to the Colombian environment aimed to help facilitate older adults to make future healthcare decisions [44]. Educational resources may support the process of advance care planning, yet available resources are not universally accepted, and they are under-utilized in clinical practice. Recently in Colombia, various healthcare insurance companies began actively promoting the formulation of written ACD which is an important step. However, this process lacks a clear communication strategy regarding the existence of ACD and their potential role, this is therefore an important area for improvement in the country.

Similarly, there are some non-governmental organization initiatives to facilitate access to legally compliant advance directives for patients and caregivers (DescLAB initiative) [45]. Unfortunately, these resources are accessible mostly through the Internet, limiting access to those with difficulties in connectivity and digital literacy.

## Conclusions

Our results suggest a lack of open conversations regarding EoL matters in patients with advanced cancer, with their physicians and family caregivers. Training in and implementation of effective communication strategies regarding EoL care for patients, physicians, and caregivers are urgently needed in Colombia.

## Supplementary Information

**Additional file 1. **Physician's questionnaire.

**Additional file 2. **Caregiver's telephone interview.

## Data Availability

The datasets used and/or analysed during the current study are available from the corresponding author on reasonable request.

## Abbreviations

EoL: End-of-life
ACD: Advance care directive
INC: Instituto Nacional de Cancerología Bogotá
HUSI: Hospital Universitario San Ignacio Bogotá
HUSJ: Hospital Universitario San José Popayán
IQR: Interquartile range
