# Supplementary material for: Knowledge of end-of-life wishes by physicians and family caregivers in cancer patients
Source: BMC Palliat Care. 2021 Sep 10;20:140. doi: 10.1186/s12904-021-00823-1 (PMC8434705; doi:10.1186/s12904-021-00823-1)
Supplement: Supplementary file 2 — Additional file 2. Caregiver's telephone interview. [file 12904_2021_823_MOESM2_ESM.docx]

**Additional file 2. Caregiver's telephone interview.**

Demographic questions

***Instructions for interviewer:***

***The interviewer should insert personal information in the questions, when appropriate. For example, use the patient's name where the word [PATIENT] appears. As I had previously explained to you, we are conducting a study to learn about the end of life of cancer patients. For this, we are contacting relatives or caregivers of patients who have died from cancer. In this interview, we would be asking you to provide information about [PATIENT NAME]. How would you like us to refer to him / her?***

**This survey consists of several parts, some about the family situation of [PATIENT], some about yourself, others about health and medical care in the last week of [PATIENT] life and finally some about the impact that the disease of [PATIENT] had in his family.**

# Each question will have some response categories, I will be informing you.

**It is important that you know that your participation is completely voluntary, and that it will be recorded. This recording will only be used by me to be able to complete some questions if necessary. Once that verification is complete, I will be deleting the recording. Your personal data or those of [PATIENT] will be recorded for the study.**

# You can decide to stop participating in the interview at any time, without any consequences for you.

**Do you have any questions?**

# Well ... we start with some questions about the situation of [PATIENT]

1. Informed consent mode

- Verbal (recorded)
- Electronic – Email
- Electronic – Verbal form (without recording)

1. Case number (equal to Study A - physicians)
2. What type of cancer had [PATIENT] been diagnosed with?


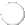
 Breast Ca


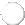
 Prostate Ca


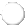
 Stomach Cancer


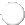


Cervical cancer
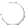
 Lung cancer


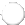

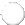
 Colon and Rectum Don't know

1. How long ago did you find out about [PATIENT] 's diagnosis?
2. How was he related to [PATIENT]?


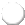

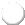
Couple

Son/daughter


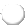


Father mother


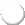
 Brother / sister

other, please specify:

1. ¿Did [PATIENT] have a partner?

*If the couple is the interviewee - do not ask but fill in "yes"*


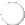
 Yes
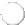


Does not Know

Not

1. [PATIENT lived with you?


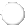
Yes


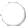


Not

1. Who (else) did [PATIENT] live with?

*Use the ¨mas¨ when the respondent answered yes to the previous question*


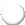
 Alone, independent


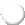
 With couple


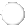

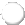
 With partner and children

Other (s), please specify


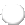
 Without a partner with children


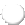
 With parents


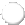
Does not know

1. Did [PATIENT] live in urban, rural, municipal or dispersed rural areas?


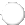
 Urban


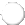
 Municipal head


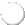
 Scattered rural


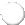
 other (specify)

1. How long ago did [PATIENT] die? For the purposes of the study it would be important to have the exact date, if you know it. *In case of not having exact date, use approximate date or option "don't know"*

Know exact date Day / Month / Year


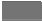
Date

DD / MM / YYYY

Know approximate date


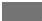
Date

DD / MM / YYYY

Don't know, place 01/01/1990


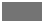
Date

DD / MM / YYYY

1. Did [PATIENT] have children?

Not


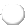


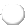
 Dont Know


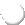
 Yes, please indicate the number ofchildren:

1. How old were your children when [PATIENT] died)? Please specify ages of all children.

Older than 30 years Between 18 and 30

Between 12 and 17

years old

Under 12 years old Does not know

1. What educational level did [PATIENT] reach?


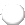
 Primary


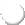
 high school


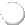
 Technical education


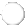
 Graduate


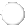
 college


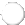
 other, please specify:

1. Did [PATIENT] die at home or in the hospital?


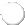
 In your house / apartment


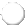

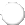
 In someone's house / apartment close to you
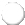
 In hospital or clinic

Other, please specify:


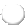
 Postgraduate – specialization


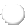
 Postgraduate - Master's


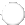

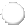
 Postgraduate - doctorate Don't know

1. Did [PATIENT] receive palliative care?


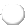
 Yes


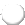
 Not


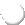
 I'm not sure

1. The hospice care was mainly home, outpatient or hospitalized?


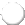
 Domiciliary


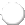
 Ambulatory


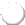
 Hospitalized


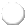
 Other, please specify

1. Was [PATIENT] treated for pain, nausea and vomiting, shortness of breath, and other symptoms at the end of life?


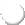
 Yes
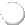
 Not


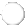
 I'm not sure


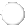
 Comments

P2: family member's information

Now, we have a few questions about yourself.

1. Do you practice any religion? If so, which one?

*Although the word "religion" is used, they can also be themes of "spirituality".*


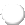
 Not


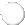

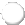
 Yes catholic
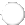
 Yes christian
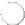
 Yes jew

Other, please specify


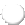
 Yes muslim


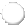
 Yes hindu


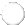
 Yes, buddhism


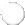
Not a specific religion,I do believe in a "God" or I am spiritual

1. How often do you attend religious services or meetings of your religious community?

*Although the word "religious" is used, they can also be themes of "spirituality".*


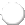
 Never


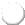
 A few times a year


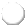
More or less monthly


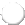
 More than once a month
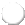
 Weekly


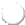
More than once a week

1. How important is religion to you?

*If indicated to be "spiritual" change to "spirituality"*


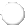

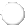
 Very important
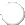
 Important

Not important

1. How involved were you in the care of [patient] in the last few weeks prior to his death?


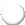
 He was very involved, one of the main caretakers


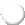
 Somewhat involved, accompanying in some processes


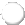
 Little involved


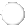
 Uninvolved


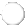
 other (specify)

1. Were you present at the time of [PATIENT] death?


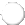
 Yes I was present


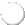
 No I was not present

1. In hindsight, would you have wanted to be present at the moment of death?


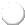
 Yes


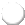
 Not


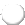
 I do not care


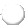
 If not, why not?

1. Was there some kind of parting with [PATIENT] before his death?


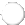
 Yes
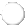
 Not

If yes, please summarize farewell

Caregivers Evaluation of Quality of End of Life Care (CEQUEL)

**Instructions for interviewer:**

***The interviewer should insert personal information in the questions, when deemed appropriate. For example, use the patient's name where the word [PATIENT] appears.***

***This instrument is designed to assess the quality of life of the patient just before death, in the hospital.***

- ***If the patient was hospitalized at the time of death, please use the phrase: “During this last week…. "***
- ***In the scenario in which the patient was not hospitalized at the time of death or in the month prior to death, ask about “the care given by (and determines who was the caregiver) during the last week of the patient's life.***

# Now we move on to a part of the interview where there will be questions about [PATIENT] 's last week of life.

1. I am going to ask you some questions about the last week of [PATIENT] life.

Where was [PATIENT] during the last week of life?


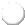
 Hospital or home care


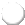
 At home or home withouthealth care


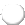
other (specify)

1. During this past week, was [PATIENT] 's life prolonged by medical interventions longer than you would have expected?

*Let the interviewee answer the question, do not give "I don't know" as an option so that the wild card does not become. Obviously, if you really don't know, use this option.*


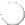

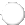
 Yes
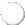
 Not


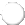
 Dont Know

comments

1. During this past week, was [PATIENT] 's life being prolonged by medical interventions when he / she was, as far as you know, already in the process of dying?


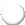
 Yes


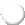
 Not


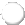
 I Dont Know


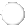
 comments

1. During this last week, was [PATIENT] 's life prolonged by the medical treatments he received and did this cause him greater suffering?


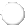
 Yes Not

I Dont Know

1. During this last week, was the information that the doctor gave you about the treatment and the process before death clear?

Yes

Not

I Dont Know

comments

1. During this past week, did the treating physician or someone from the medical team who treated [PATIENT] talk with him / her about his / her preferences in terms of medical treatment?

Yes Not

I dont know

1. Did [PATIENT] express any wishes about medical treatment at the end of life?

*They can be formal or informal advance directives or verbal expressions about your wishes.*

I did have an advance directive

Yes, informally

No, he did not express his wishes

I dont know

Other (specify) / space for comments

1. During this last week, was there any medical procedure or treatment that was applied to him / her that was inconsistent with his / her previously stated wishes or that he / she would not have wanted?

Yes

Not

I I dont know

If so, please comment on the treatment or the unwanted situation

1. During this past week, did the doctors you talk to about treating [PATIENT] listen to your concerns about it?

Yes Not

I I dont know

1. During this past week, did you or your family receive any information about what to expect while [PATIENT] was passing away?

Yes Not

I dont know

1. Would you have appreciated receiving any additional information about what to expect while [PATIENT] was passing away?

Yes

Not

other (specify)

1. Did you or your family ever receive information about medications that would be used to manage pain, shortness of breath, or other symptoms from [PATIENT]?

Yes

Not

I I dont know

Comments

1. Would you have liked (more) information about medications?

Yes N ot

1. During this last week, how often were you or your family members informed about the situation / condition of [PATIENT]?

Forever

Frequently

Sometimes

Never

I don't know / other: specify

Nature of Death

**Nature of the Death (NAT)**

In the next section of the survey, we are going to ask a few things related to the time of death of [PATIENT]. The answers are on a scale of 1 to 7 points. In each question I will be informing you about the score on the scale. For each question, please mention the number that best describes how you feel.

1. From 1 to 7, how much do you think [PATIENT] suffered when he died?

Minimum Moderate Very much

Suffering

other (specify)

1. From 1 to 7, in your opinion, how calm or traumatic did [PATIENT] death seem to you?

Peaceful Moderate Traumatic

Death

other (specify)

1. From 1 to 7, how much did [PATIENT] suffer compared to what you would have expected?

A lot less Same A lot ofmore

Suffering

other (specify)

Quality of Death and Dying (QODD)

Survey for relatives on the end of life of an oncology patient

**I am going to ask you some aspects of the last week of [PATIENT] life. Now the scale changes a bit: the questions have answers on a scale from "never" to "all the time", with "never" being one and "all the time" being 5 points on the scale. There are no right or wrong answers, the important thing is that you provide us with your impression of the situation of [PATIENT] in his last week of life.**

1. From 1 to 5, how often did [PATIENT] seem to have his pain controlled?

Never Enoughtimes All the time

Pain control

other (specify)

1. From 1 to 5, how often did [PATIENT] have control over what was happening in their environment?

Never Enoughtimes All the time

Control over what was happening in your environment

other (specify)

1. From 1 to 5, how often was [PATIENT] able to feed himself?

Never Enoughtimes All the time

Feeding alone

other (specify)

1. From 1 to 5, how often did [PATIENT] have control of his sphincters, both urine and bowel movements?

Never Enoughtimes All the time

Toilet training

other (specify)

1. From 1 to 5, how often was [PATIENT] able to breathe without much difficulty?

Never Enoughtimes All the time

Difficulty breathing

other (specify)

1. From 1 to 5, how often did [PATIENT] seem uneasy about death?

Never Enoughtimes All the time

Peace of mind in the face of death

other (specify)

1. From 1 to 5, how often did [PATIENT] seem not afraid of dying?

Never Enoughtimes All the time

Not be afraid of dying

Don't know or other (specify)

1. From 1 to 5, how often did [PATIENT] smile or laugh in those last few days?

Never Enoughtimes All the time

Laughed and smiled

other (specify)

1. From 1 to 5, how often did [PATIENT] have the energy to do what they wanted?

Never Enoughtimes All the time

Energy to dowhat i wanted

other (specify)

1. From 1 to 5, how often did [PATIENT] seem to be concerned about overloading their loved ones or becoming a nuisance to them?

Never Enoughtimes All the time

Burden for your loved onesdear

other (specify)

1. From 1 to 5, how often did [PATIENT] seem to maintain his dignity and respect?

Never Enoughtimes All the time

Dignity and respect

other (specify)

1. From 1 to 5, how often did [PATIENT] spend time with a spouse or partner (or did not have a partner)?

Never Enoughtimes All the time

Time with spouse or partner

I did NOT have a partner or other (specify)

1. From 1 to 5, how often was [PATIENT] with your children? (or had no children)

Never Enoughtimes All the time

Frequency withchildren

I had no children or another(specify)

1. From 1 to 5, how often did [PATIENT] spend time with friends and other family members?

Never Enoughtimes All the time

Time with friends and other family

other (specify)

1. From 1 to 5, how often did [PATIENT] spend time alone?

Never Enoughtimes All the time

Time alone

other (specify)

1. [PATIENT] had pets? If yes, how often did [PATIENT] spend time with pets (or had no pets)?

Never Enoughtimes All the time

Time with pets

I had no pets or other (specify)

1. Was [PATIENT] able to find meaning and meaning in his life?

Yes Not

I dont know

1. Was [PATIENT] accompanied and hugged by those he loved?

Yes Not

I dont know

1. Was [PATIENT] able to attend important events (graduations, weddings, birthdays)?

Yes Not

I dont know

Comments observations:

1. Did [PATIENT] have all health costs covered?

Yes Not

I dont know

1. Was [PATIENT] able to say goodbye to loved ones?

Yes to everyone Yes, to the closest but not to all

Yes to some

NoT

I dont know

1. Did [PATIENT] have one or more visits from a religious or spiritual advisor?

Yes Not

I I dont know

1. Did [PATIENT] have a spiritual rite or ceremony before passing away?

Yes Not

I dont know

1. Did [PATIENT] use a mechanical ventilator (or breathing machine) or dialysis to prolong his life?

Yes Not

I dont know

1. Was [PATIENT] able to heal bad feelings?

Yes Not

I dont know

1. Did [PATIENT] have the funeral arrangements in order before passing away?

Yes, costs were covered (insurance)

Yes, he had expressed how he wanted thefuneral arrangements

Yes, both costs andthe arrangements were in order

Not

I dont know

1. Was [PATIENT] able to discuss his wishes regarding the care of the dying process with his doctors and others?

Yes Not

I dont know

1. Was anyone present at the time of [PATIENT] death?

Yes Not

I I dont know

1. At the time of death, [PATIENT] was ...

Awake

Sleeping

In a coma /Inconscious

Does not know

Does not respond

COVINSKY FAMILY IMPACT SURVEY

Survey for relatives on the end of life of an oncology patient

# I would like to ask you about some aspects in which [PATIENT] 's illness may have affected other members of the family.

1. Has anyone in your family gotten sick or had stress or tension problems associated with [PATIENT] illness and death that caused you to stop doing your daily activities?

Yes Not

I do not know

Comments

1. Did [PATIENT] illness mean having to use all or most of the family's savings?

Yes Not

I do not know

Comments

1. Did [PATIENT] 's illness mean the loss of the main source of income for your family?

Yes Not

I do not know

Comments

1. Did the costs associated with care for [PATIENT] disease cause the family to move to a less expensive place to live?

Yes Not

Comments

1. Did the costs associated with care for [PATIENT] necessitate postponing important medical care for someone else in the family?

Yes

Not

I do not know

Comments

1. Did the costs associated with care for [PATIENT] require postponing education plans or significantly changing plans for any other family member?

Yes Not

Dont Know

Comments

1. Please tell me more about these changes (skip if not applicable):

Interview closing

Survey for relatives on the end of life of an oncology patient

CLOSING QUESTIONS

We would like to know how you felt participating in thisinterview

1. Did you find participating in this interview disturbing or difficult?

Not at all or verylittle Something disturbing

Moderately disturbing

Quite disturbing Very disturbing

1. To what extent do you feel this interview is beneficial / useful for you?

Very beneficial

Moderately beneficial

Little beneficial

It had no benefit

1. Do you have any other comment about your participation in this interview?

What is the gender of the person who answered this survey?

Questions for interviewer

Survey for relatives on the end of life of an oncology patient

Male

Feminine

1. What was the general attitude of the interviewee during the questions?

Friendly

Cooperative but not very friendly Indifferent

Suspicious

Hostile and uncooperative

1. What was the level of general emotional affectation of the interviewee?

Minimal or no affectation

Moderately affected

Affected

Very affected

1. Please rate the confidence of the interviewee in providing information.

Highly reliable

Pretty reliable

Trustworthy

1. Was the entire interview completed?

Yes

Not

Survey for relatives on the end of life of an oncology patient

1. If it was not completed in full, please indicate why:

Fatigue

Emotional affectation

Time extension

Other, please specify:

1. Space for comments or notes from the interviewer about this interview
